# Hour-Glass Contraction Previous to the Delivery of the Child

**Published:** 1880-03

**Authors:** J. R. Moore

**Affiliations:** Member of Committee on Obstetrics, New London, Wis.


### ARTICLE III.

HOUR-GLASS CONTRACTION PREVIOUS TO THE DELIVERY OF THE CHILD. Paper read before the Northwestern Wisconsin Medical Society in December, 1879. By J. R. MOORE, M.D., Member of Committee on Obstetrics, New London, Wis.

Nov. 1, 1879.—Called at eight o'clock, a. m. to see Mrs M——, in confinement. Nativity, American; in stature above the medium size and very muscular; age, forty years and pregnant with fourth child.

Had been sick the greater part of the night, and first stage terminated about six o'clock, a. m. with a very abundant discharge of liquor amnii, after which pains became regular and severe at intervals of about ten minutes.

On making an examination, I found the os well dilated, soft and flabby, and the head, which was presenting, seemed determined to force its way in front of and to the right of the symphysis pubis (right iliac region). The abdominal walls were flabby, owing to the loss of tonicity from the large abundance of water so recently discharged, and uterine contractions powerful.

My first impression was, that with a few pains I would be able to dislodge the head and bring it within the axis of the superior strait. This I set myself about, and, between pains, I could bring it so that it seemed that it would engage itself within the brim of the pelvis; but, directly as the pain returned, it would return to its former position.

And for three long hours, I thus, with my fingers, tried to change the position of the head, but in vain.

The pains now had become so severe that during them I was obliged to allow the inhalation of chloroform.

Externally, the outlines of the head could be distinctly felt in the right iliac region and even slight pressure occasioned great pain. Waiting till eleven o'clock, three hours from the time I first saw her, and seeing no advancement whatever, I made another attempt with the vectis to change the position of the head, which I could do between pains, as before, but directly as pain returned, back it would go.

Of course, every advantage was taken of position and every external appliance imaginable made use of, such as bandages, counter-pressure with the hands over the tumor, etc., but all in vain; and at two o'clock, p. m., eight hours from the termination of the first stage, there being no change whatever, only a more exhausted condition of the patient, I called in Dr. P. Dickinson, who lived near by, in consultation.

After making an examination, the doctor concluded (as I had thought) that time would overcome the evil and force the head within the brim of the pelvis. So, with a determination to succeed, he went to work as I had done for so long a time; and thus he worked for two hours, with the same result. At the end of which time the patient showed great signs of exhaustion, with a quick, sharp pulse, lips dry and parched, tenderness increasing rapidly on pressure over abdomen, muttering delirium when slightly under the influence of chloroform and pains still frequent and very hard. Making an examination again, and finding no change in the presenting part, I was now firm in the belief that some other alternative must be resorted to, and that at once or our patient would die before delivery could be effected. In this the doctor agreed with me, and we concluded to try and grasp the head between the blades of Hodges' long forceps and see if we could not effect a delivery by that means. But with the presentation *occipito-iliac* this was an utter impossibility.

Failing in this, which of the other two alternatives must be resorted to, viz, craniotomy or version? The condition of the patient seemed to favor the former method, while the general impression would be to turn. And this we fortunately concluded to do. So, placing the patient in the proper position, viz, on her back, and allowing the free inhalation of chloroform, I proceeded to find the feet. The pelvis was roomy and the soft parts offered but little resistance, also the uterus until I passed the head. But here I found as great a constriction as is possible to find in hour-glass contraction after delivery of the child—the secret of the whole mischief.

Below the constriction there seemed to be but little muscular contraction, but at and above, it was indeed powerful. I at once began to dilate this constriction, but it was so great that it was with difficulty that I could pass the end of my index finger under it. You can rest assured that it was no simple matter to dilate it sufficiently to admit the passage of the hand. But this I at last accomplished, secured a foot, and, after some little difficulty, succeeded in turning and delivering a living boy, weighing eight and a half pounds, being smaller than any of her children before it. After delivery of the child, hour-glass contractions again followed, which we were obliged to overcome to remove the placenta. But this was not difficult, as there was no unnatural attachment between it and the womb. It was attached near the fundus.

This is the first instance where I have known this form of contraction to exist before the delivery of the child, and indeed I can find nothing in obstetrical literature which bears directly upon this point. Cazeaux, Meigs, Velpeau, Byford and others dwell at length upon *irregular* contraction, etc., but say nothing of this form, which may exist, as is here proven, to such extent as to require surgical interference to effect a delivery.

And is it not possible that we may have this difficulty to deal with many times when we are not aware of its true nature? As in this case, if we had resorted to any other method of procedure, as craniotomy, or the use of the instruments if the head could have been grasped, we might have met with great difficulty in removing the body of the child from above the constriction. But if it had been accomplished, we would never have been the wiser as to the true cause for surgical interference.

But why not have hour-glass contraction *before*, as well as *after* the delivery of the child, if there is an opportunity for it? And what I mean by an opportunity is this, viz., suddenly changing the womb from a *highly distended* to an *empty* condition, or in other words, from a condition where the walls are extremely tense, to one of flaccidity, as in this case, when the liquor amnii was discharged, as there was a superabundance of it. I was not present at the time, but the attendants declared that there was no less than a pailful.

I am firm in the belief that, without this sudden change in the condition of the womb, this form of contraction will not manifest itself, having no faith in the theory that any attachment of the placenta can exert any influence whatever in that direction.

But let the cause be what it may, we can only theorize as to that. But we know that it may give rise to a very unpleasant state of affairs.

And ought we not to look carefully for this obstacle when surgical interference seems necessary, and try to overcome it before resorting to a more hazardous undertaking?

NEW LONDON, WIS., Jan. 30, 1880.

